# Switchable Adhesion of Hydrogels to Plant and Animal Tissues

**DOI:** 10.1002/advs.202411942

**Published:** 2024-12-07

**Authors:** Leah K. Borden, Morine G. Nader, Faraz A. Burni, Samantha M. Grasso, Irene Orueta‐Ortega, Mahima Srivastava, Paula Montero‐Atienza, Metecan Erdi, Sarah L. Wright, Rajabrata Sarkar, Anthony D. Sandler, Srinivasa R. Raghavan

**Affiliations:** ^1^ Department of Chemical & Biomolecular Engineering University of Maryland College Park MD 20742 USA; ^2^ Fischell Department of Bioengineering University of Maryland College Park MD 20742 USA; ^3^ Sheikh Zayed Institute for Pediatric Surgical Innovation Children's National Medical Center Washington DC 20010 USA; ^4^ Division of Vascular Surgery University of Maryland Baltimore MD 21201 USA

**Keywords:** Adhesion on demand, adhesion strength, reversible adhesion, smart materials, Tissue microstructure

## Abstract

The ability to “switch on” adhesion between a thin hydrogel and a biological tissue can be useful in biomedical applications such as surgery. One way to accomplish this is with an electric field, a phenomenon termed electroadhesion (EA). Here, it is shown that cationic gels can be adhered by EA to *tissues across all of biology*. This includes tissues from animals, including humans and other mammals; birds; fish; reptiles (e.g., lizards); amphibians (e.g., frogs), and invertebrates (e.g., shrimp, worms). Gels can also be adhered to soft tissues from plants, including fruit (e.g., plums) and vegetables (e.g; carrot). In all cases, EA is induced by a low electric field (DC, 10 V) applied for a short time (20 s). After the field is removed, the adhesion persists. The adhesion can also be reversed by applying the field with opposite polarity. In mammals, EA is strong for many tissues (e.g., arteries, muscles, and cornea), but not others (e.g., adipose, brain). Tissues with anisotropic structure show anisotropic adhesion strength by EA. The higher the concentration of anionic polymers in a tissue, the stronger its adhesion to cationic gels. This underscores that EA is mediated by the electrophoresis of chain segments across the gel‐tissue interface.

## Graphical abstract

Electroadhesion (using 10 V DC for 30 s) can be used to stick cationic hydrogels to animal and plant tissues (which are anionic). After the field is removed, the adhesion persists, but applying the field with opposite polarity reverses the adhesion. This phenomenon can be used to create unusual biohybrid materials as well as to perform sutureless surgery.

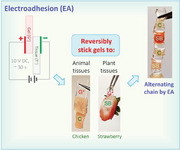

## 1 Introduction

The ability to achieve adhesion “on command” between two soft materials is an interesting and valuable property. Consider two water‐containing solids that do not adhere when contacted. An external stimulus is then applied, whereupon strong, persistent adhesion is induced (switched on) between the pair. While various studies have used light or magnetic fields as the switch,^[^ ^1^, ^2^ ^]^ the stimulus of interest in this work is *electric fields*.^[^ ^3^, ^4^, ^5^, ^6^, ^7^, ^8^, ^9^ ^]^ We are particularly interested in the case where one solid is a hydrogel, i.e., a 3‐D network of crosslinked polymer chains in water, and the other a biological tissue. In an earlier limited study, we showed that cationic gels could be adhered to some bovine tissues by an electric field.^[^ ^8^ ^]^ A gel and a tissue that did not stick to each other were contacted and 10 V DC was applied across the pair for ∼20 s. Thereupon, a strong, enduring adhesion, termed electroadhesion (EA), was induced between the pair.^[^ ^8^ ^]^ Only cationic gels (denoted by G^+^) could be adhered by EA to tissues, not anionic or nonionic gels. Interestingly also, EA could be reversed simply by reversing the polarity of the DC field and applying the same 10 V DC for ∼30 s. Thus, the adhesion induced by EA was inducible on‐demand, strong and persistent, and yet reversible.

Our findings raise intriguing possibilities, but also numerous questions. The ability to affix a gel over a tissue on command could enable a new way to perform surgery: i.e., a new way to seal tears or cuts in human tissues without using sutures.^[^ ^8^ ^]^ But can G^+^ gels be adhered by EA to tissues from humans or other mammals? Can EA be achieved in live animals? And how about animals other than mammals? Also, why did EA work with some bovine tissues and not others? This also brings up the mechanism for EA: what is its origin? Mammalian tissues are generally known to have anionic character (and hence are denoted here on by T^−^). The fact that only G^+^ gels adhere by EA to T^−^ tissues implies a need for oppositely charged polymer chains across an EA interface. EA may then involve the electrophoresis of chain segments across this interface, leading to strong electrostatic bonds.^[^ ^3^, ^4^, ^5^, ^6^, ^7^, ^8^, ^9^ ^]^ In the context of gel‐tissue adhesion, another question is whether gels and tissues are similar in their structure. Should we view a tissue as cells embedded in an extracellular matrix (ECM), with the ECM being a polymer network? If not, what kind of structure is needed for a tissue to undergo EA?

Here, we test the switchable adhesion of cationic G^+^ gels via EA to a range of biological tissues. A number of surprising and intriguing findings are reported for the first time. We show adhesion to tissues from *across the animal kingdom* (including humans, other mammals, birds, fish, reptiles, insects, worms, etc.). We also show adhesion of gels to various *soft tissues from the plant kingdom*, including those from vegetables and fruits. Together, our results imply that adhesion of gels by EA is a *universal feature across all of biology*. We have measured the adhesion strength induced by EA using pull‐off testing. Consistent trends are found with regard to tissue type: for example, muscles in different animals (cow, chicken, mouse, fish) exhibit EA adhesion strengths around 15 kPa. Also, tissues with anisotropic structure adhere stronger to gels in one orientation (transverse) over the perpendicular one (longitudinal). Gels can be adhered by EA not only to soft tissues like the liver but also to stiff ones like cartilage. Our results shed insight into the mechanism for EA, the structure of biological tissues, and the clinical potential for EA in surgery.

## 2 Results and Discussion

### 2.1 Electroadhesion Works the Same Way for Various Pairs of Soft Materials

**Figure 1:**
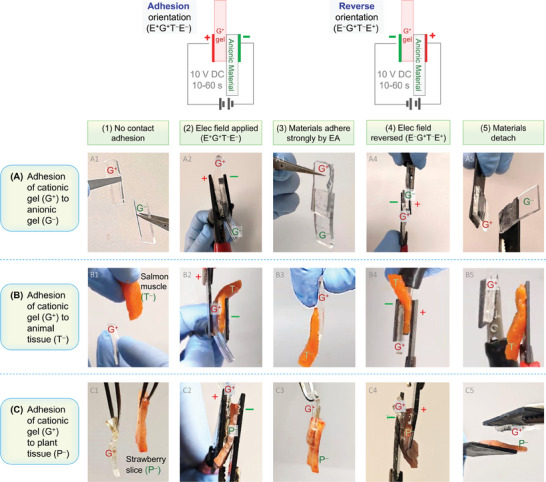
Common features in the electroadhesion (EA) of three material pairs. EA requires a cationic and an anionic material. Here, the cationic material is a QDM gel (G^+^). Adhesion of this gel is shown via photos to: A) an anionic gel (G^–^) of SA; B) anionic animal tissue (T^–^, muscle from salmon); C) anionic plant tissue (P^–^, slice of strawberry). (1) The material pairs exhibit no adhesion on contact. (2) A DC field of 10 V is turned on for ∼30 s in the adhesion orientation (i.e., E^+^G^+^T^–^E^–^). (3) Upon removal from the field, the materials are strongly and permanently adhered. (4) The adhered pair is placed in the same DC field in the reverse orientation (i.e., E^–^G^+^T^–^E^+^) for ∼30 s. (5) Upon removal from the field, the materials have lost their adhesion and can be detached.

We will first demonstrate EA between three pairs of soft materials. In each pair, one material is cationic while the other is anionic. The cationic material is fixed to be a hydrogel denoted chemically as QDM and symbolically as G^+^. This gel is made by free‐radical polymerization of a mixture containing the cationic monomer quaternized dimethyl aminoethyl methacrylate (QDM).^[^ ^8^, ^9^ ^]^ The three panels of **Figure** **1** show that QDM (G^+^) gels can be adhered by EA to a variety of anionic materials, including anionic gels (Figure 1A), animal tissues (Figure 1B), and plant tissues (Figure 1C). The anionic gel (G^−^) is made by polymerizing a mixture containing the anionic monomer sodium acrylate (SA).^[^ ^9^ ^]^ As an example of an animal tissue (T^−^), we use a slice from a species of fish, i.e., salmon. This slice corresponds to the muscle, and it exhibits a vivid orange color. As an example of plant tissue (P^−^), we use a slice of strawberry. Both the animal and plant tissue slices in Figure 1 are extracted from their inner portions, avoiding their exterior skin. The anionic nature of these tissues will be discussed in a later section.

All materials in Figure 1 are cut into thin strips with a thickness between 2 to 5 mm and a long dimension ∼2 cm. They are all soft solids with sufficient dimensional integrity such that they can be lifted and held vertically between one's fingers. Prior to performing an EA experiment, as a first step, we tested the three pairs of materials (G^+^/G^−^, G^+^/T^−^, and G^+^/P^−^) for contact adhesion. That is, we pressed each pair together by hand and observed if the materials remained adhered. In all cases, contact adhesion was minimal, and the materials could be easily separated (this is indicated in the vertical Panel 1, i.e., Photos A1, B1, C1). The lack of contact adhesion underscores the fact that the subsequent adhesion (i.e., EA) is due to the electric field.

Next, we connect the materials to a DC power source in the orientation necessary to induce EA (Panel 2). In this orientation, the cationic gel (G^+^) contacts the positive electrode (E^+^) and the anionic material (e.g., T^−^) the negative electrode (E^−^).^[^ ^3^, ^8^ ^]^ In this E^+^G^+^T^−^E^−^ orientation (see schematic above Panel 2), we apply a DC voltage of 10 V for a short time (between 10 to 60 s). During this time, the material pairs develop a strong adhesion, which is what we term electroadhesion (EA). The EA develops within 10 s for the G^+^/G^−^ pair, while it takes ∼30 s for the G^+^/T^−^ pair and ∼60 s for the G^+^/P^−^ pair. The field is then turned off and each pair is found to be strongly adhered (Panel 3) — the pairs are held vertically in Photos A3, B3, and C3 to demonstrate their persistent adhesion. Importantly, the EA is maintained indefinitely thereafter (e.g., gel samples adhered by EA have stayed adhered for years).^[^ ^3^, ^8^ ^]^ Next, we show that the adhesion is reversible. The adhered pairs are placed back in the DC electric field, but the orientation is reversed (Panel 4): the cationic gel (G^+^) now contacts the negative electrode (E^−^) and the anionic material (e.g., T^−^) contacts the positive electrode (E^+^). While in this E^−^G^+^T^−^E^+^ orientation (see schematic above Panel 4), we apply the same 10 V DC again for 10 to 60 s. When the field is turned off, the pairs are found to have lost their adhesion and can be easily detached.^[^ ^3^, ^8^ ^]^

To summarize, we reiterate some of the common features of EA across the range of materials in Figure 1:

1. No contact adhesion is observed.
2. Adhesion is induced by applying a DC electric field (10 V) in a specific “adhesion” orientation (i.e., E^+^G^+^T^−^E^−^).
3. The electric field is applied for a short time (≈10 to 60 s).
4. The adhesion persists forever after the field is switched off.
5. The adhesion can be reversed by applying the same DC potential in the “reverse” orientation (i.e E^−^G^+^T^−^E^+^).
6. In all cases, adhesion is between a cationic and an anionic material.

All these features are observed in every case of EA documented in this paper. In the literature, other instances of electrically triggered adhesion are described (and even termed “electroadhesion”), but they are mostly between two hard materials or a hard and a soft material (not two aqueous soft materials like gels and tissues).^[^ ^10^, ^11^, ^12^, ^13^, ^14^, ^15^ ^]^ They also either require much higher voltages, an AC field, or for the field to be applied over long times (e.g., > 20 min). Moreover, such adhesion is usually lost soon after the field is removed.

### 2.2 Gels can be Electrodhered to Tissues from Animals Across the Entire Animal Kingdom

**Figure 2:**
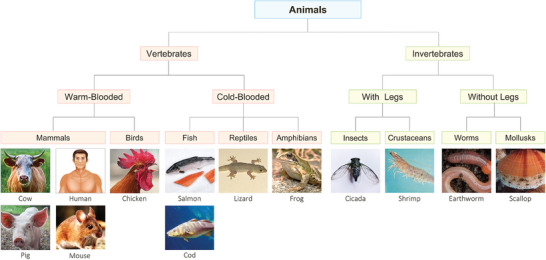
Cationic gels can be adhered by EA to tissues across the animal kingdom. EA of QDM gels (G^+^) is accomplished to a tissue from each of the animals in this chart (see further details in Figure S1, Supporting Information). Each animal is placed in its appropriate phylum and class. All EA tests were done using 10 V DC applied for 30 s.

We now will discuss EA of cationic (G^+^) gels to tissues of various animals (**Figure** **2**). In previous work, we had shown that these gels could be adhered by EA to bovine (cow) tissues only.^[^ ^8^ ^]^ In this paper, we have tested tissues from animal species extending across the various phyla and classes.^[^ ^16^, ^17^ ^]^ All the animals in Figure 2 show evidence of EA to at least one of their tissues, indicating that the phenomenon of gel‐tissue EA extends across the animal kingdom. EA was done using the same conditions discussed under Figure 1: a DC field of 10 V was applied across the gel‐tissue pair for 30 s. The field was then switched off and the gel‐tissue pair was assessed visually. If strong adhesion occurred, the pair could be held vertically as shown in Figure 1, Panel 3 and the materials would not detach. Moreover, when adhesion was strong, attempts to dislodge the gel from the tissue using tweezers would be unsuccessful and the gel would break in the process. In selected cases, we also measured the strength of adhesion using pull‐off testing. These measurements confirmed that our visual assessments tallied with appreciable values for the adhesion strength (to be discussed in a later section). We also confirmed that: (a) the adhesion induced by EA was much stronger than contact adhesion; and (b) the adhesion could be reversed by reversing the polarity of the field.

We will now discuss the various animals in Figure 2. Among warm‐blooded vertebrates,^[^ ^16^, ^17^ ^]^ we found EA to tissues of mammals (including human, cow, pig, mouse) and birds (chicken) — these results are further classified by tissue type and discussed in Section 2.4. Among cold‐blooded vertebrates,^[^ ^16^, ^17^ ^]^ we found EA to tissues of fish (salmon and cod), amphibians (frog), and reptiles (lizard). EA also occurs with selected tissues in invertebrates^[^ ^16^, ^17^ ^]^ that are land‐based (insects like cicada and worms like the earthworm) or aquatic (crustaceans like shrimp, and mollusks like the scallop). For the non‐mammals, details on the specific body parts to which EA was done are given in Figure S1 (Supporting Information). Note that in animals with a hard shell, EA was achieved to the soft interior. It was only in the cases of frogs and earthworms that EA could be directly achieved to the outer skin (Figure S1, Supporting Information): these are animals with a soft body and without a hard exterior shell. We emphasize that every animal we tested had a tissue to which a G^+^ gel could be attached by EA. There were no exceptions to this rule.

### 2.3 Gels can be Electroadhered to Tissues from Plants

**Figure 3:**
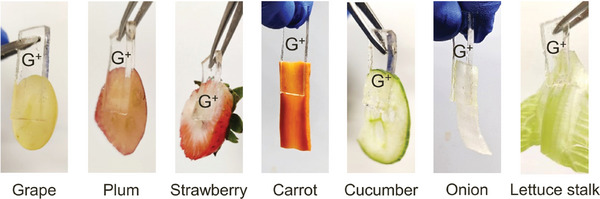
Cationic gels can be adhered by EA to various materials derived from plants. Photos are shown of QDM gels (G^+^) adhered by EA to the insides of different fruits and vegetables. All EA experiments were done using 10 V DC applied for 30 to 60 s.

In addition to the animal kingdom, we also tested tissues from biological materials belonging to other kingdoms, specifically plants. Successful EA of cationic (G^+^) gels was accomplished with plant‐based materials (**Figure** **3**). The conditions were the same as for animals (10 V DC for 30–60 s). The examples include^[^ ^16^ ^]^ root vegetables (carrot), leafy vegetables (lettuce), bulb vegetables (onion), vegetables and fruits that grow on vines (cucumber, grape), fruit that grow on trees (plums), and fruit from herbs (strawberries).

Note from the photos in Figure 3 that the gel is adhered to the inner portion of the vegetable or fruit and not to its outer skin (the skin is generally a hydrophobic layer)^[^ ^18^, ^19^ ^]^. In the case of lettuce, EA only occurred with the stalk and not the leaves. We did try EA to the exterior layer of many plant leaves and flower petals — with no success. Again, a hydrophobic coating is expected to be present on the surfaces of leaves or flowers.^[^ ^18^ ^]^ Additionally, no EA was found in the cases of some fruit (pineapple, peach, melon) and vegetables (tomato, radish). Thus, unlike with animals, there are a few plant‐based materials that do not permit adhesion by EA, at least under the conditions studied here.

### 2.4 Electroadhesion Varies with Animal Tissue Type

**Figure 4:**
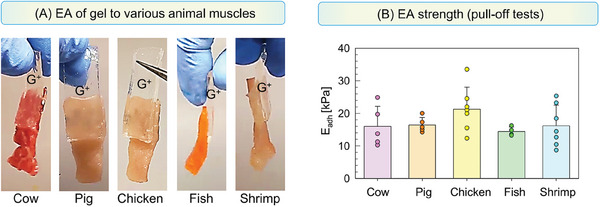
Adhesion of cationic gels by EA to skeletal muscles from different animals. A) Photos of QDM gels (G^+^) adhered by EA to skeletal muscles (transverse segments) from various animals. All EA experiments were done using 10 V DC applied for 30 s. B) Adhesion strength *E*_adh_ from pull‐off tests for the various pairs in (A). For each pair, *n* ≥ 5 measurements were done and all the data points are shown. The error bars correspond to the standard deviations.

We now discuss results for various tissue types in animals. One tissue type that exists in mammals, birds, fish, and even invertebrates like crustaceans is the muscle.^[^ ^16^, ^17^ ^]^ We adhered cationic (G^+^) gels to muscles from several animal species using EA under the typical conditions (**Figure** **4**). The species tested included mammals (cow, pig, mouse), birds (chicken), fish (salmon), and crustaceans (shrimp). In all cases, the muscle chosen was a skeletal muscle and the segment selected was in the transverse orientation (this will become important later). All pairs show strong adhesion, which is evident from the photos in Figure 4A. This adhesion was further quantified by pull‐off tests (see Experimental Section for details). The adhesion strength *E*_adh_ plotted in Figure 4B is the stress required to pull the gel off the tissue. All values of *E*_adh_ for the various gel‐muscle pairs are around 17 kPa. Thus, the data confirm that gels can be strongly adhered to muscle tissue regardless of the species from which the muscle is taken. In each of the above cases, we also confirmed that the *E*_adh_ induced by EA was much higher than *E*_adh_ for simple contact between gel and tissue. Soft materials often show weak adhesion on contact due to the ability of their surfaces to conform to each other when pressed together.^[^ ^20^, ^21^ ^]^ The strength of this contact adhesion can vary depending on the contact area (i.e., the surface roughness) as well as the softness (viscoelasticity) of the materials. For our gel‐tissue pairs, this contact adhesion typically corresponded to *E*_adh_ < 5 kPa.

**Figure 5:**
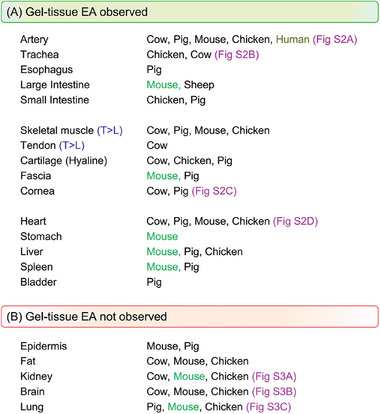
Classification of tissues regarding their ability to adhere to cationic gels by EA. The classification is based on EA experiments (10 V DC for 30 s) done with a given tissue and a QDM gel (G^+^). For each tissue type, tissues were sourced from different animals. Experiments with live mice are indicated in green. Details are shown for selected tissues in Figures S2 and S3 (Supporting Information).

Next, we turn our attention to other tissues relevant to mammals. We have been able to test multiple tissues for EA from four mammals (cow, pig, sheep, mouse) and one bird species (chicken). We can thereby identify similarities in EA across species, as shown in **Figure** **5**. Here, tissues are divided into two categories based on whether or not they adhere to G^+^ gels by EA. Tissues that adhere to gels are grouped in Figure 5A: all these tissues exhibit an *E*_adh_ induced by EA that is higher than contact adhesion. Tissues that do not adhere to gels by EA are grouped in Figure 5B: in all these cases, the adhesion was either non‐existent or not distinguishable from contact adhesion. Further details (with photos) of gel‐tissue EA experiments are provided in Figures S2 and S3 (Supporting Information) for selected tissues. Typically, a thin slice was cut from the exterior surface of the tissue, and EA to a similar slice of G^+^ gel was attempted under the usual conditions (i.e., 10 V DC for 30 s). In Figure 5, entries are also shown for mice. These experiments were done in many cases with live mice, and if so, the entry is color‐coded in green. Details on these experiments will be reserved for a separate paper.

The list in Figure 5A of tissues that adhere to gels encompasses many of the important tissues in our body. These include arteries, intestines, trachea, and esophagus, all of which are tubular tissues that convey either blood, air, or digested food.^[^ ^16^, ^17^ ^]^ The *E*_adh_ of G^+^ gels to bovine aorta was measured to be 47 kPa. We also studied EA to a human cadaveric femoral artery allograft, which is typically used as a conduit for bypass surgery: *E*_adh_ for this case was measured to be 21 kPa. Note that the *E*_adh_ values for a given tissue type can vary considerably with species. Other adhering tissue types include muscle, tendon, cartilage, fascia, and cornea. For muscle and tendon, the orientation of the tissue influences adhesion, which is disussed below under Figure 7. Organs that show adhesion include the vital organs such as the heart, stomach, liver, spleen, and bladder. The liver and spleen were two organs that consistently showed weaker adhesion to G^+^ gels compared to tissues like skeletal muscles or arteries. However, in general, if the adhesion due to EA to a given tissue was weak, it could be enhanced by increasing the voltage (e.g., from 10 to 15 V) or the application time (e.g., from 30 to 90 s). These effects of voltage and time on gel‐tissue adhesion have been discussed in our earlier paper.^8^

The tissues in Figure 5B did not show adhesion to gels, neither under the usual conditions (10 V, 30 s), nor at higher voltages or application times. These are hence the outliers in our studies, and we wanted to see if they shared any common features. Fat (adipose) tissue is unique among all the ones in Figure 5 in that it has a low water content (≈15%) and a high content of non‐polar (oily) fat.^[^ ^22^ ^]^ All other tissues have a water content of 60% or higher. As a result, the current measured in an EA experiment with fat is close to zero. In the cases of brain, kidney, and lung, although their water contents were high, we again found that the currents measured in our EA experiments were very low (see Figure S3, Supporting Information). Thus, a low current seems to be one common factor for the tissues in Figure 5B. Current in gels and tissues is carried by ions,^[^ ^23^, ^24^ ^]^ and hence a low current may indicate that the concentration of ions (and in turn, the ionic conductivity) may be too low in these tissues.

### 2.5 Presence of Anionic Biopolymers in Tissues May Hold the Key to Electroadhesion

**Figure 6:**
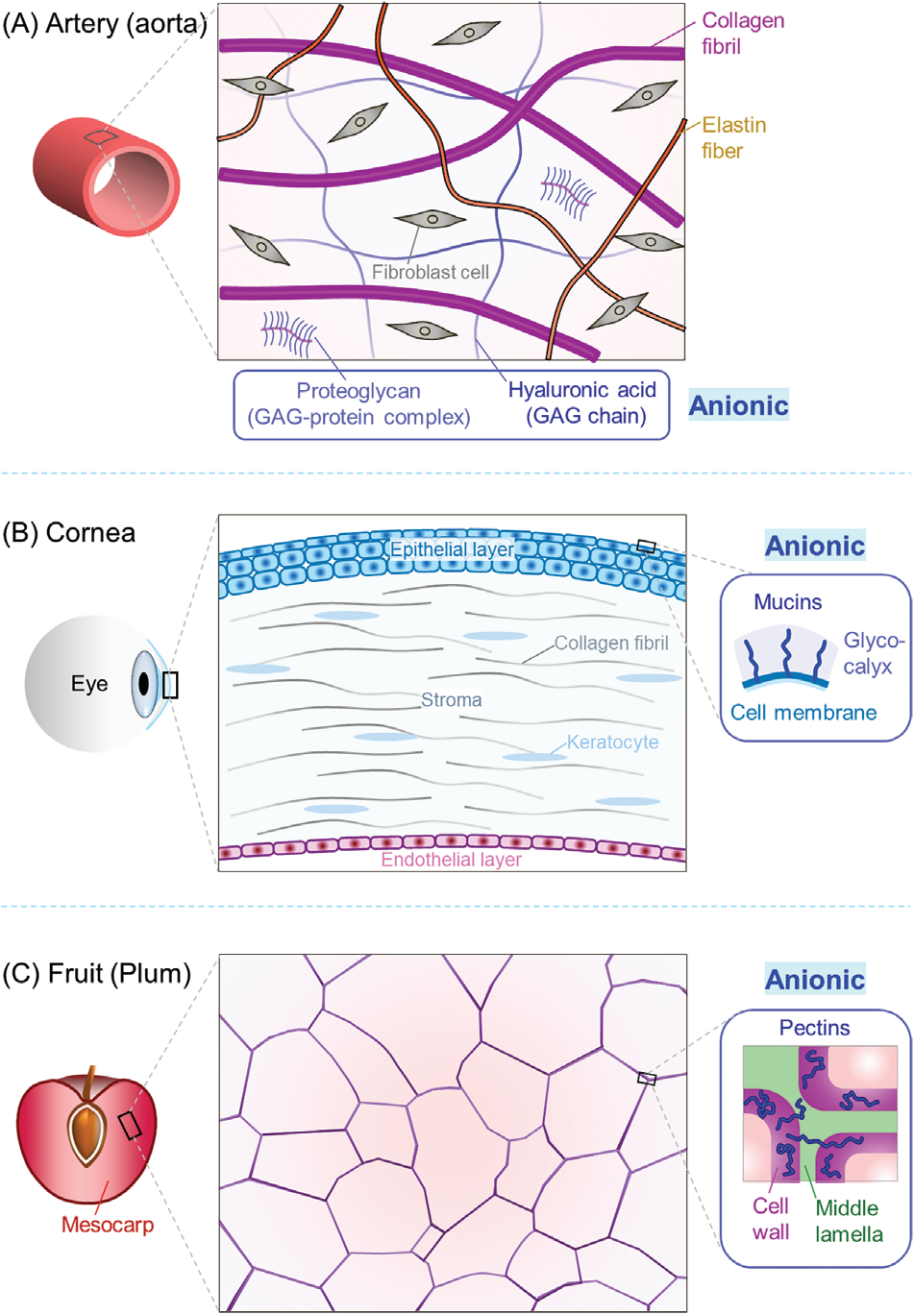
Structures of animal and plant tissues to which gels can be adhered by EA. In all cases, anionic biopolymers are expected to be present in the tissue. A) The outer layer of the aorta has cells embedded in an extracellular matrix (ECM), which contains anionic glycosaminoglycans (GAGs).^[^ ^17^ ^]^ B) The outer layer of the cornea has close‐packed epithelial cells. Each cell membrane extends out an anionic glycocalyx.^[^ ^28^ ^]^ C) Plant tissues are made of close‐packed cells, each having a cell wall. The anionic polysaccharide pectin is present in the cell walls as well as in the middle lamellae between cells.^[^ ^30^ ^]^

Why do some tissues show EA to gels while others do not? This is a complex question, but as a first step to deciphering it, we must discuss the microstructure of biological tissues. All living creatures are composed of cells (microscale containers).^[^ ^16^, ^25^ ^]^ In multicellular organisms such as animals and plants, cells are organized into tissues, with each tissue type having distinct functions. Tissues are macroscale solids with dimensional stability. They are composed of cells and an extracellular matrix (ECM).^[^ ^16^, ^25^ ^]^ The ECM is a network of biopolymers secreted by the cells. In terms of their structure, tissues fall into two types, as shown by the schematics in **Figure** **6**: 1) Cells embedded within an ECM; and 2) Cells close‐packed and connected into a solid with very little ECM.^[^ ^16^, ^25^ ^]^ An example of (1) is the external layer of arteries like the aorta (Figure 6A). Two examples of (2) are shown, one from animals (the cornea, Figure 6B) and the other from plants (plum interior, Figure 6C). The chosen examples all exhibit EA to G^+^ gels. They are further discussed below.

**Figure 7:**
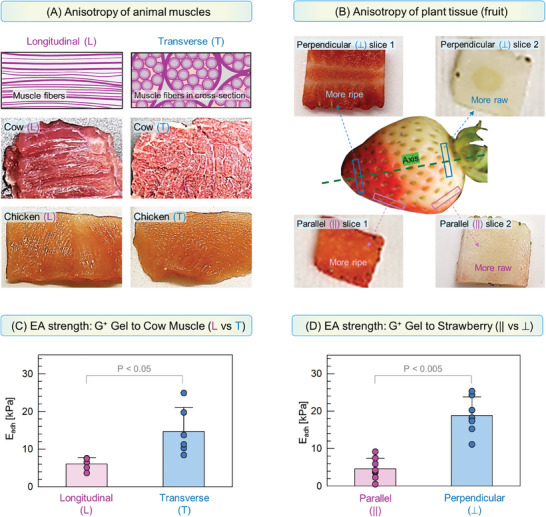
Tissue anisotropy and its effects on adhesion to cationic gels by EA. A) In the case of animal skeletal muscle, a transverse (T) cut through the muscle exposes fiber tips, which are absent in a longitudinal (L) cut. The differences between the two cuts can be seen even at the macroscale in photos of cow and chicken muscle. B) In the case of plant tissues (strawberry), thin slices perpendicular to the axis of the fruit (⊥, shown in blue) are distinct in regards to EA from slices parallel to the axis (||, shown in pink). This is true regardless of whether the tissue is raw or ripe. (C,D) show results for tissues adhered to QDM gels (G^+^) by EA (10 V, 30 s). The adhesion strength *E*_adh_ from pull‐off testing is plotted. (C) For cow muscle tissues, *E*_adh_ is much higher for the T segments. (D) For strawberry tissues, *E*_adh_ is much higher for the ⊥ slices. For each data point in (C,D), *n* ≥ 4 measurements were taken and all the data points are shown. Error bars correspond to standard deviations.

In the first category of tissue, consider the outer layer of the aorta (tunica externa),^[^ ^17^, ^26^ ^]^ which is a connective tissue consisting of cells (fibroblasts) embedded in an ECM (Figure 6A). The ECM here is a disordered fibrous mesh akin to a polymer network in a hydrogel. Thick fibers of collagen (∼20 µm diameter), thin elastic fibers of elastin (∼1 µm diameter), and polymer chains (∼1 nm diameter) called glycosaminoglycans (GAGs) are all present in the ECM, as shown in the schematic.^[^ ^27^, ^28^ ^]^ The GAGs are all anionic and either exist as linear chains (e.g., hyaluronic acid, HA) or in a comb‐like architecture as part of proteoglycans (PGs). A key point is that the GAGs give the tissue an anionic character. The same is largely true of most connective tissues, including the cartilage and tendon.^[^ ^27^ ^]^ In some connective tissues like the tendon, the concentration of fibers is very high and these fibers may be aligned, but the anionic nature due to the GAGs is always present. Muscles are another class of tissue where cells are in a fibrous form, and the anionic bioopolymer there is the protein actin. We will discuss muscles in **Figure** **7**.

In the second category of tissues (formed by close‐packing of cells), we mention the cornea, which is the thin (0.5 mm), transparent front part of the eye (Figure 6B).^[^ ^17^, ^29^ ^]^ It has a top (apical) layer of epithelial cells and a bottom layer of endothelial cells. As shown in the schematic, the cells in these layers pack tightly to form the tissue. More generally, epithelial and endothelial layers of all tissues are formed by close‐packing of cells.^[^ ^17^ ^]^ Epithelial cell membranes of the cornea extend out a layer of membrane‐bound glycoproteins and glycolipids. This layer is termed the glycocalyx and it projects ∼500 nm into the surrounding tear fluid.^[^ ^29^, ^30^ ^]^ Many of the glycoproteins are anionic, including transmembrane mucins and proteoglycans. Their anionic nature keeps the eye hydrated and helps to fight infection. Below the epithelial layer, the stroma is a layer that contains cells (keratocytes) in an ECM (aligned collagen fibers and GAGs). The stroma can be up to 90% of the cornea. Thus, the cornea has multiple layers, two formed by close‐packing of cells and the middle stroma consisting of cells in an ECM. We observe strong adhesion of G^+^ gels via EA to the corneal surfaces.

Lastly, in the case of plant tissue (Figure 6C), such as in plums or strawberries, the soft tissue (mesocarp) is again formed by close‐packed cells, each having a cell wall in addition to a cell membrane.^[^ ^31^, ^32^ ^]^ The cell walls consist of polysaccharides, specifically cellulose and pectin. Cellulose is uncharged and is in the form of microfibrils within the cell wall. Pectin is an anionic polysaccharide that is present both within the cell wall as well as in the space between adjacent plant cells called the middle lamella (up to 2 µm in thickness).^[^ ^33^, ^34^ ^]^ Pectin thus acts as a bridge between plant cells. It is often found complexed with calcium (Ca^2+^) ions.

To summarize, animal and plant tissues both have a net anionic charge (which is why we use T^−^ and P^−^ to denote them). The anionic nature is imparted by different types of biopolymers in the different tissues: glycoproteins like the GAGs (in connective tissues) or mucin (in the ocular glycocalyx); proteins like actin (in muscle); or polysaccharides like pectin (in plant cell walls). Anionic biopolymers are present in the ECM in some tissues (e.g., aortal outer layer); or attached to cell membranes (e.g., corneal epithelium); or embedded in cell walls (e.g., fruit cells). The density of anionic charge is likely to be the first key factor in explaining EA variability across tissues. That is, we hypothesize that strong adhesion to G^+^ gels by EA arises when a *high fraction of anionic polymers* is exposed at the tissue surface. Conversely, a low fraction of anionic polymers at the surface may explain the weak to no adhesion of the G^+^ gel with other tissues. Note that a lack of ionic polymers (and hence of counterions) may also explain the low currents observed for the tissues in Figure 5B.

To understand the importance of anionic biopolymers, it is useful to place in context the prevailing mechanism for EA. Previous instances of EA were mostly between cationic (G^+^) and anionic (G^−^) gels.^[^ ^3^, ^7^, ^9^ ^]^ A schematic of EA between two such gels is shown in Figure S4 (Supporting Information). Here, the gels are shown as crosslinked networks of chains with ionic backbones. When the electric field is applied in the adhesion orientation (E^+^G^+^G^−^E^−^), cationic chain segments between crosslinks are believed to undergo electrophoresis and migrate into the anionic gel (across the interface).^[^ ^3^, ^7^, ^9^ ^]^ Anionic chain segments will do the reverse and migrate into the cationic gel. In turn, these chain segments at the interface will strongly bind by electrostatic interactions. When the field is switched off, this binding will persist, which is why the gels remain adhered. If a tissue is anionic (T^−^) and has a structure similar to an anionic gel (G^−^), then the analogy perfectly applies and we can understand how EA to a G^+^ gel can arise in a similar way.^[^ ^8^ ^]^ This may indeed be the case for a tissue in which cells are embedded in an ECM (Figure 6A). For tissues formed by close‐packed cells, the anionic chains may be part of (or anchored to) the cell membrane or wall (Figure 6B,C). Can these chains undergo electrophoresis similar to chains of the ECM? This seems plausible, but it is an open question that requires further study in the future. Two other factors related to charge density are discussed next.

### 2.6 For a Given Tissue Type, Electroadhesion May Vary with Tissue Orientation

As briefly mentioned earlier under Figure 5, EA results for gel‐muscle adhesion are markedly different for transverse or longitudinal segments. We believe these differences are related to the anisotropy of muscle tissues.^[^ ^17^, ^35^ ^]^ Figure 7 discusses tissue anisotropy in more detail, and we see its effects on EA in both animal and plant tissues. In the case of animal (skeletal) muscle (Figure 7A), the tissue consists of densely packed fibers arranged in aligned bundles (myofibrils).^[^ ^17^, ^35^ ^]^ The fibers are formed from the proteins actin and myosin, with the anionic actin being the major component (thereby making the tissue anionic). A longitudinal (L) cut of the muscle will be parallel to the fibers and will thus not expose the fiber tips. Conversely, a transverse (T) cut of the muscle will sever the fibers and thus expose the fiber tips.^[^ ^17^, ^35^ ^]^ Both the L and T segments can be adhered to G^+^ gels, but the data in Figure 7C show that the pull‐off adhesion strength *E*_adh_ is much higher to the T than the L segments (15 kPa versus 6 kPa). We believe this is because the surface of the T segment exposes a higher density of anionic proteins (i.e., actin) compared to the L segment.

Anisotropy in plant tissues is illustrated in Figure 7B in the case of a strawberry. A slice of strawberry can be cut either perpendicular to its axis (blue rectangles) or roughly parallel to its axis (pink rectangles). When tested for EA with G^+^ gels (Figure 7D), the former exhibits much higher *E*_adh_ than the latter (20 kPa vs 4 kPa). These differences are surprising — and it does not matter if the strawberry is ripe (red colored) or raw (white colored). In fact, the anisotropy of plant tissues has a known origin.^[^ ^30^, ^31^ ^]^ For these tissues to grow, the cell wall has to disintegrate. The anionic pectin in the cell wall is the key for enabling this disassembly. Cell growth is dictated by an enzymatic process that converts the carboxylate in pectin to an ester. Esterified pectin loses its charge and thus cannot crosslink with Ca^2+^; in turn, the cell wall is weakened, and the tissue grows due to turgor pressure in the direction of the weakened cell wall. In other words, plant tissues will typically have a high fraction of anionic pectin in one orientation (the growth direction) and esterified (nonionic) pectin in the other orientation (the mature direction).^[^ ^32^ ^]^ Thus, the orientations of the strawberries we examined may again correspond to different pectin fractions, with the higher pectin correlating with a higher *E*_adh_.

The larger point is that biological tissues, both animal and plant, can be both anisotropic as well as inhomogeneous. Anisotropy means that the same tissue has different properties along different axes. It arises due to the microstructure of the tissue — e.g., if aligned fibers are present as in the case of skeletal muscle (Figure 7A). Note here that the two orientations of muscle (L and T) can have identical chemical composition in the bulk, and yet may expose different extents of anionic polymers at their surface. Tissues can also be inhomogeneous with respect to chemical composition itself. Taking the case of a mammalian tissue like the aorta (an artery that proceeds from the heart to other parts of the body), its composition close to the heart can be different from that further away. These points must be kept in mind when we assess the capacity of a given type of tissue to undergo EA. When comparing the same tissue from different mammals in Figure 5, we have tried to ensure that the analyzed segments correspond to roughly the same region in the animal.

### 2.7 Electroadhesion is Observed with Both Soft and Stiff Tissues

Having found EA to many animal and plant tissues, we wondered if the rheological (mechanical) properties of either the gel or the tissue was a crucial factor for adhesion. When two soft materials are brought into contact, their viscoelastic nature (i.e., their compliance) can induce adhesion.^[^ ^20^, ^21^ ^]^ That is, adhesion can arise when compliant gel and tissue surfaces interlock and fill in each other's crevices. Is such “viscoelastic adhesion” a key to EA? We regard this as unlikely because there are controls built into every EA experiment. That is, we first ensure that there is negligible adhesion on contact (Control 1) and also that the EA‐induced adhesion can be undone by applying the field with reverse polarity (Control 2). If visoelasticity was a factor, it should affect these controls, i.e., the gel and tissue should adhere in the absence of the field. It is unlikely for the rheology of the gel (or tissue) to be altered by applying a modest electric field (10 V) for a short time (at most 60 s). All the results in this paper are for cases where EA far exceeds contact adhesion and hence we do not consider viscoelasticity to underlie the EA effect.

Even if viscoelasticity is not a critical factor, we can still wonder if rheology can help explain some of our results. For instance, does the G^+^ gel adhere more strongly by EA to tissues that are soft? Or ones that are stiff? Is adhesion stronger when the gel and tissue have similar stiffness? To address such questions, we measured the dynamic rheological properties of both the G^+^ gel and various animal and plant tissues. For all samples, the elastic (*G*′) and viscous (*G*″) moduli from oscillatory shear were measured as functions of the angular frequency *ω*.^[^ ^21^ ^]^ As expected, all gels and tissues exhibited an elastic response, with *G*′ > *G*″ over the range of *ω* and with both moduli being nearly independent of *ω*.^[^ ^13^, ^21^ ^]^ The value of the elastic modulus *G*′ (at a fixed *ω* of 10 rad s^−1^) can then be taken as a convenient measure of the tissue stiffness.

We measured the stiffness *G*′ of our standard G^+^ gel to be 14 kPa. An animal tissue with comparable stiffness is skeletal muscle from the chicken, with its *G*′ being 15 kPa. Stiffer tissues include chicken cartilage (*G*′ = 110 kPa), while softer tissues include chicken liver (*G*′  = 3 kPa). As indicated in Figure 5A, gel‐tissue EA is found for all the above, i.e., the *gel adheres to tissues that are both stiffer and softer than itself*. However, we did not find a clear trend connecting tissue stiffness (*G*′) and EA strength (*E*_adh_). We did note earlier that EA to liver and spleen (two soft tissues) is rather weak. To test if this adhesion could be improved by using softer gels, we made a G^+^ gel with a lower amount of chemical crosslinker. A gel with *G*′ = 3 kPa was tested for EA with chicken liver, which has a matching *G*′. However, the resulting *E*_adh_ was indistinguishable from that of the original G^+^ gel. Likewise, we also made a stiffer G^+^ gel (by increasing the crosslinker) and tested it with a stiff tissue like cartilage: again there was no difference in *E*_adh_ when compared to the original G^+^ gel. Thus, matching the stiffness of the gel and tissue does not seem important for EA.

**Figure 8:**
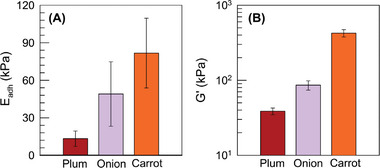
Adhesion of cationic gels by EA to plant tissues of varying stiffness. A) Pull‐off adhesion strength for G^+^ gels adhering by EA to three plant tissues. B) Elastic modulus (G’) from dynamic rheology for the three tissues. For each data point, at least three samples were measured. Error bars correspond to standard deviations.

We did unearth a pattern when examining the EA between our G^+^ gel and plant tissues (**Figure** **8**). The pull‐off adhesion strength *E*_adh_ is plotted in Figure 8A for three such tissues: plum, onion, and carrot. *E*_adh_ for the case of plum is reasonably high (13 kPa) and is comparable to that for skeletal muscle in Figure 4B. But *E*_adh_ for onion is even higher (49 kPa) and the highest *E*_adh_ is found with carrot (82 kPa). Indeed, the gel‐carrot EA is the strongest adhesion among all pairs we have tested for this paper. Intuitively, we expect carrot to be a stiff tissue and much stiffer than plum, and this is borne out by the *G*′ values plotted in Figure 8B. The *G*′ of carrot is 420 kPa, which is much higher than that of most animal tissues we have studied. In comparison, both onion and plum have lower *G*′, with the *G*′ of plum (39 kPa) being comparable to that of animal tissues. Thus, on the whole, our results show that EA of the same gel can be achieved to both stiff tissues (*G*′ > 400 kPa) as well as soft tissues (*G*′ < 4 kPa). Overall, this spans a 100‐fold range in tissue stiffness.

Why does the EA adhesion strength seem to correlate with stiffness for plant tissues? And why is such a trend not seen with animal tissues? We believe animal tissues can be very different from each other: for example, their anionic nature can stem from a variety of polymer chemistries (GAGs, glycoproteins, or proteins). The tissues can also have different microstructures (aligned fibers or disordered networks of chains) (see Figures 6 and 7). Due to these differences, it is difficult to compare different types of animal tissues. Plant tissues, on the other hand, have a similar structure of close‐packed cells (Figure 6C) and the anionic component in all cases appears to be pectin. Thus, when comparing plant tissues that are soft (e.g., plum) versus stiff (e.g., carrot), the difference is likely to correlate above all with the *total polymer concentration* in the tissue. In other words, carrot will have a higher polymer content than plum (or equivalently, a lower water content than plum). If the anionic (pectin) fraction is about the same in both, this means that there will be a *higher concentration of anionic chains* in carrot than in plum. This can explain the higher *E*_adh_ seen with G^+^ gels.

### 2.8 Some Closing Thoughts on Electroadhesion

In Sections 2.5 to 2.7, we have identified three factors that can dictate the EA adhesion strength of a given tissue to cationic gels. First, the tissue must have polymers with strong anionic character: the higher the **fraction** of such anionic polymers, the higher the *E*_adh_. Second, the anionic polymers must be prevalent and **accessible** at the tissue interface, which is why the anisotropic structure of tissues influences *E*_adh_, as shown by Figure 7. Third, for a given tissue type, the higher the **concentration** of anionic polymers, the higher the *E*_adh_, as shown by Figure 8. Note a caveat to the third point: if the polymer concentration in the tissue becomes too high, then, in turn, the water content may drop to very low (≈10% to 30%) levels. This is the case with the leaves or stems of plants as well as fat tissues in animals. If so, the ionic conductivity of the tissue may become too low for an electric current to pass through — and then EA is not observed.

Analogous points apply to the cationic gel used for gel‐tissue EA. In this work, we have used the same cationic gel (QDM) in all studies and it is the gel we had used previously.^[^ ^8^, ^9^ ^]^ But for a given tissue, we can stick a variety of cationic gels by EA. Indeed, *any cationic gel* with a high concentration of cationic chains can be used. The chemistries of four different cationic gels that work for EA are shown in Figure S5 (Supporting Information). We make our QDM gel by polymerizing a monomer mixture of acrylamide (AAm) and QDM in a molar ratio of 100:16 (≈6:1) (Figure S5A, Supporting Information). The crosslinks in this gel are chemical (covalent) bonds. Instead of QDM, we can use other cationic monomers to make similar chemical gels (Figure S5B, Supporting Information). Alternatively, we can create cationic gels using cationic biopolymers such as chitosan (Figure S5C, Supporting Information) or cationic guar (Figure S5D, Supporting Information). These can be crosslinked by covalent bonds (e.g., chitosan with glutaraldehyde) or physical bonds (e.g., the guar with borax, where the crosslinks are hydrogen‐bonds). *Both chemical and physical gels can be stuck to tissues by EA*. While a full comparison of all these gels is beyond the scope of this paper, we note that gel‐tissue EA appears to be the strongest in the case of QDM gels. This is likely because QDM has a strong and pH‐insensitive positive charge from its quaternary ammonium group.

**Figure 9:**
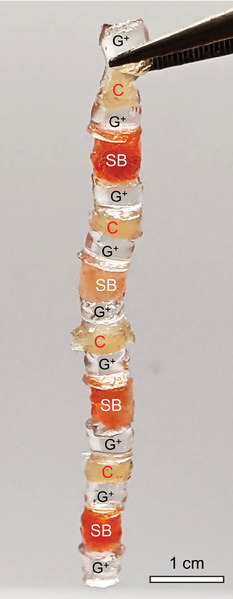
Gel‐tissue chain made by EA. Pieces of a cationic gel (G^+^) and anionic tissues (chicken (C) and strawberry (SB)) are connected in a chain by EA without the use of any adhesive or glue.

Finally, to illustrate the new possibilities afforded by EA, **Figure** **9** shows an unusual chain made by this technique. Here, we have alternated pieces of a cationic gel (G^+^) with pieces of chicken (C) and strawberry (SB). All pieces had diameters around 5 mm. For each gel‐tissue pair, EA (10 V DC, 20 s) was used to induce adhesion. While adhering the next pair in the chain, the previous gel‐tissue interfaces were left in open circuit so that their adhesion would not be undone by the field.^[^ ^9^, ^13^ ^]^ This is similar to the strategy we used in our earlier paper to make chains of cationic and anionic capsules.^[^ ^9^ ^]^ The resulting chain is robust and withstands modest deformation. It can be viewed as a novel biohybrid of gel, plant and animal tissues.

## 3 Conclusion

In this work we investigated the EA of cationic gels with a range of biological tissues. We fixed the cationic gel composition and the EA conditions (10 V, 10 to 60 s) and varied the tissues. In all cases of EA documented in this paper, the adhesion induced by EA was much stronger than contact adhesion (i.e., prior to applying the field) and moreover, the EA adhesion persisted indefinitely after the field was switched off. The adhesion could also be undone later by applying the same DC potential with reversed polarity. EA of the cationic gel was found to tissues from various animal species, including mammals, birds, fish, reptiles, amphibians, insects, and worms. Similar EA was also found with plant tissues, including fruits and vegetables. To our knowledge, this is the first report of EA for most of the tissues listed above. We broadly conclude that adhesion of cationic gels by EA is **universal across the range of soft tissues encountered in biology**.

Because of the potential for EA to be used in surgery, we paid particular attention to the EA of cationic gels to mammalian tissues. From studies across a range of mammals (cow, pig, mouse, chicken, human), we found EA with the majority of tissue types, including the arteries, muscles, and cornea. However, negligible adhesion was observed in the cases of adipose, brain, and lung. Three factors correlated with the EA adhesion strength *E*_adh_, measured by pull‐off testing. The first was that the tissue needed to have strongly anionic polymers. Examples of putative polymers include hyaluronic acid (in the ECM of animal tissues); mucins (in the glycocalyx around many animal cells); and pectin (in the cell walls of plant cells). Second, the anionic polymers needed to be accessible at the tissue interface with the gel — accordingly, if a tissue was anisotropic, *E*_adh_ varied with tissue orientation. Third, for some tissues such as those from plants, a higher concentration of anionic polymers implied a higher *E*_adh_ (and also a higher tissue stiffness). Thus, we have identified significant trends that shed insight into both the EA phenomenon as well as the structure of biological tissues.

Our findings also have clinical implications for the use of EA in surgery. We have found EA to a number of solid organs (e.g., liver, spleen) where bleeding may have to be arrested rapidly — and this could be attempted via EA of a gel patch. Moreover, we have also found EA to a number of tubular organs (blood vessels, intestines, trachea), which can undergo tears or even a complete rupture. Direct sutureless repair of these tubes using gels is a promising application of EA in surgery, and it is actively being evaluated by us as well as other research groups.

## 4 Experimental Section

### Materials

The following chemicals were from Sigma–Aldrich: the monomers acrylamide (AAm), sodium acrylate (SA), and 2‐(di‐methylamino)ethyl methacrylate (DM); the crosslinker *N,N’*‐methyl‐enebis(acrylamide) (BIS); the initiator ammonium persulfate (APS); the polymer chitosan (low molecular weight, 50–190 kDa); and phosphate buffered saline (PBS) in tablet form. The monomer quaternized dimethylaminoethyl methacrylate (QDM) (other names: 2‐(di‐methylamino)ethyl methacrylate, quaternary ammonium salt or [2‐(methacryloyloxy)ethyl] trimethyl ammonium chloride) was also from Sigma–Aldrich and it was a 75 wt.% solution in water. The polymer cationic guar gum (guar hydroxypropyl trimethylammonium chloride) was purchased from Making Cosmetics. The accelerant N,N,N’,N’‐tetramethylethylene‐diamine (TEMED) was from TCI America. A cyanoacrylate‐based glue (Krazy Glue) and Rust‐Oleum hydrophobic coating were purchased from The Home Depot. Deionized (DI) water was used in all experiments.

### Gel Synthesis

Cationic QDM gels were prepared by a protocol similar to that reported previously. First, DI water was degassed by bubbling nitrogen for 20 min. In 20 mL of this water, the following were combined: 1 m (1.4 g) AAm, 0.16 m (809 µL) of QDM solution, 0.019 m (0.06 g) BIS, 0.0088 m (0.04 g) APS and 0.01 m (30 µL) TEMED. The above mixture was poured into a Petri dish coated with a hydrophobic layer (Rust‐Oleum) for easy gel removal. The Petri dish was placed under nitrogen for 1.5 h, whereupon the gel became fully polymerized. Anionic SA gels were prepared in a similar manner. In this case, 1.4 m (2 g) AAm, 0.11 m (0.2 g) SA, 0.019 m (0.06 g) BIS, 0.0088 m (0.04 g) APS, and 0.01 m (30 µL) TEMED were mixed in 20 mL of degassed DI water. Polymerization under nitrogen was done for 20 min. Cationic DM gels were prepared just like the QDM gels with the DM monomer substituting for QDM at the same molar ratio. After polymerization, gels were stored in a fridge.

### Procurement of Animals and Tissues

Cow, pig, and chicken tissues were obtained ethically, immediately after slaughter from a local butcher or farm. (For the case of mice alone, see below.) Fish (salmon and cod), shrimp, and scallop were purchased fresh from Whole Foods. A lizard and a frog were obtained from a local pet owner after they had expired of natural causes. An earthworm and an insect (cicada) (both of which are invertebrates) were found in natural settings in the campus. Expired cryopreserved human femoral artery was graciously provided by Lifenet Health. All experiments on tissues were conducted within 24 h of tissue harvest. Animals/tissues were kept refrigerated from harvest point until the time of the experiment, whereupon they were brought to room temperature and then tested.

### Tissue Preparation

In the cases of most mammalian or bird tissues, when first received, organs were typically encased in fat and other matrix material. The fat and matrix material were removed to isolate the tissue of interest. This has been discussed in our previous paper,^[^ ^8^ ^]^ and examples are also provided in the Supporting Information of this paper (Figures S2 and S3, Supporting Information). The cleaned tissues were then cut into smaller pieces for the EA experiments. Typical tissue sections were cut to a thickness of around 3 mm.

### Electroadhesion Experiments

A DC power source (Agilent, model E3612A) with a range of 0–60 V, 0–0.5 A was used for the EA studies. A voltage of 10 V was used in all cases and this was applied for 10 to 60 s. Graphite electrodes (from Saturn Industries) were cut to strips of 2 × 3 × 0.15 cm, and these were connected to the power source using alligator clips. The electrodes were placed on either side of the gel‐gel pair or gel‐tissue pair, as shown in Figure 1. Gel strips were generally 2‐mm thick, while tissue strips were 2 to 5 mm thick. For the chain shown in Figure 9, samples were cut using a punch biopsy with diameter of 5 mm. Graphite needles were used as electrodes and were placed across the pair to be adhered, while leaving all other gel‐tissue interfaces in open circuit. This was similar to the procedure used previously to make a capsule chain by EA.^[^ ^9^ ^]^

### Electroadhesion Experiments with Mice

Experiments with C57 black mice were done at Children's National Hospital. All protocols used with the mice were approved by the Institutional Animal Care and Use Committee (IACUC; #30 703). During testing of live mice, the animals were under anesthesia, following an end‐point from the same study. In testing EA of a gel strip with a given tissue in the mouse (e.g., the large intestine), graphite electrodes were placed on the gel and on the tissue, and a voltage of 10 V was applied for 30 s. Adhesion was evaluated by pulling on the gel to remove it from the underlying tissue. Further details on these studies will be reported in a separate paper. After testing EA with tissues of interest, the animals were sacrificed. Some organs were then harvested from these animals for further EA studies.

### Pull‐Off Testing

A TA Instruments Q800 dynamic mechanical analyzer (DMA) was used to perform the pull‐off tests. A pair of samples (e.g., a gel and a tissue), each 3 mm in thickness and with a flat cross‐section (0.7 × 1.3 cm), were either electroadhered or adhered by contact. The remaining faces of the sample‐pair were adhered by Krazy Glue to the DMA clamps, with the tissue face to the bottom (movable) clamp and the gel face to the top (fixed) clamp. The bottom clamp was then pulled apart at a rate of 0.1 N min^−1^ until failure, and the stress at failure was recorded. Five replicates were done for each pair.

### Rheology

Rheological experiments were performed on an AR2000 stress‐controlled rheometer (TA Instruments). Samples were run on a parallel plate geometry (20 mm diameter). Dynamic frequency spectra were conducted in the linear viscoelastic regime of the samples, as determined previously from dynamic stress sweeps.

### Statistics

The data presented in Figures 4, 7, and 8 were collected and used without any transformation or normalization. At least three samples were tested for each data point. No outliers were excluded. Mean values are shown in the plots and error bars correspond to standard deviations. Statistics were calculated and plotted using Excel and SigmaPlot.

## Conflict of Interest

The authors declare no conflict of interest.

## Supporting information



Supporting Information

## Data Availability

The data that support the findings of this study are available from the corresponding author upon reasonable request.
