# Supplementary material for: Switchable Adhesion of Hydrogels to Plant and Animal Tissues
Source: Adv Sci (Weinh). 2024 Dec 7;12(5):2411942. doi: 10.1002/advs.202411942 (PMC11792046; doi:10.1002/advs.202411942)
Supplement: Supplementary file 1 — Supporting Information [file ADVS-12-2411942-s001.pdf]

ADVANCED SCIENCE
Open Access

Supporting Information

for *Adv. Sci.*, DOI 10.1002/advs.202411942

Switchable Adhesion of Hydrogels to Plant and Animal Tissues

*Leah K. Borden, Morine G. Nader, Faraz A. Burni, Samantha M. Grasso, Irene Orueta-Ortega, Mahima Srivastava, Paula Montero-Atienza, Metecan Erdi, Sarah L. Wright, Rajabrata Sarkar, Anthony D. Sandler and Srinivasa R. Raghavan**

Supporting Information for:

Switchable Adhesion of Hydrogels to Plant and Animal Tissues

Leah K. Borden,[1] Morine G. Nader,[1] Faraz A. Burni,[1] Samantha M. Grasso,[1] Irene O. Ortega,[1] Mahima Srivastava,[1] Paula M. Atienza,[2] Metecan Erdi,[1] Sarah L. Wright,[3] Rajabrata Sarkar,[4] Anthony D. Sandler,[3] and Srinivasa R. Raghavan[1,2]*

[1]Department of Chemical & Biomolecular Engineering, University of Maryland, College Park, MD 20742, USA
[2]Fischell Department of Bioengineering, University of Maryland, College Park, MD 20742, USA
[3]Sheikh Zayed Institute for Pediatric Surgical Innovation, Children's National Medical Center, Washington, DC 20010, USA
[4]Division of Vascular Surgery, University of Maryland, Baltimore, MD, 21201, USA

*Corresponding author. Email: sraghava@umd.edu

Contents

Figure S1: Details of EA experiments with various animal species.

Figure S2: Details of EA experiments with mammalian tissues that exhibited adhesion.

Figure S3: Details of EA experiments with mammalian tissues that did not exhibit adhesion.

Figure S4: Mechanism hypothesized for EA between cationic and anionic materials.

Figure S5. Chemistries of various cationic gels that can be adhered to tissues by EA.

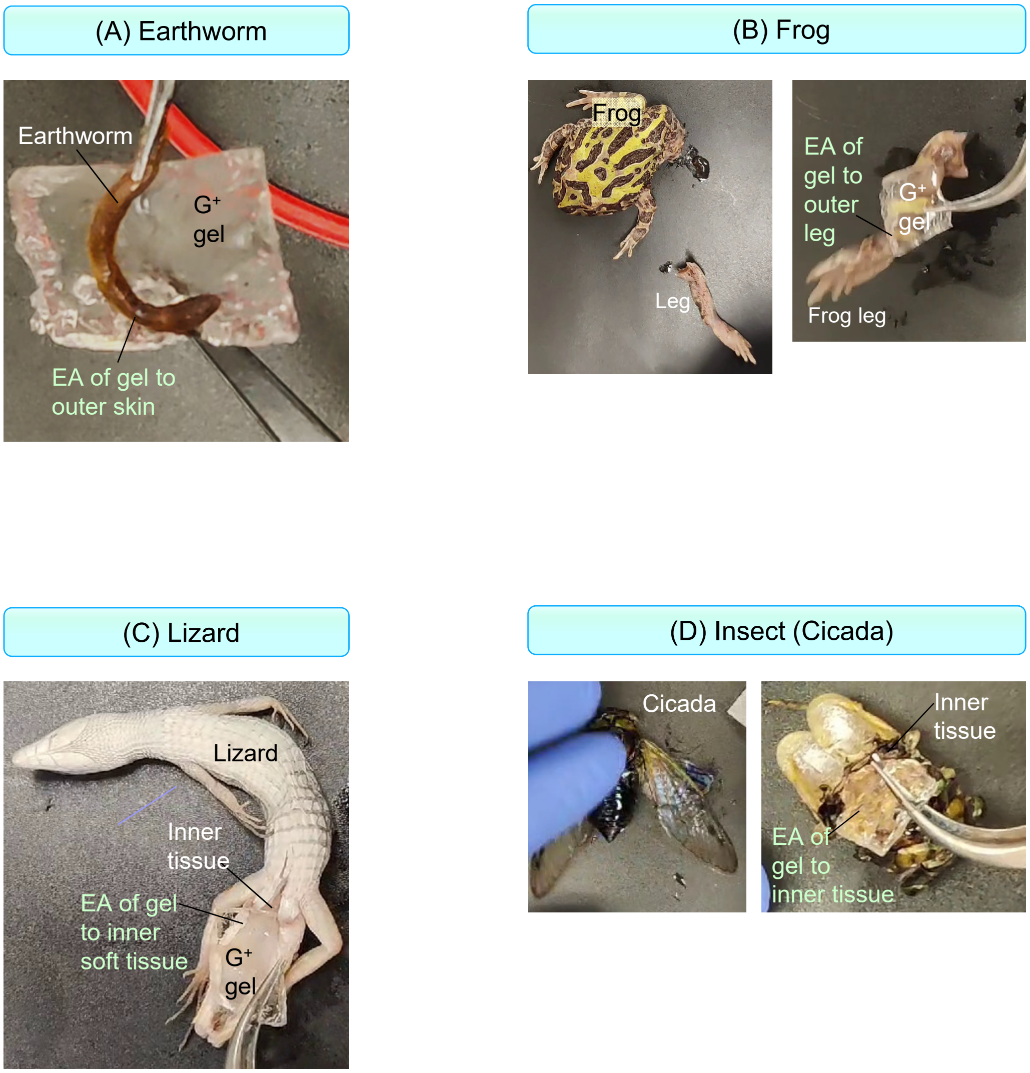

Figure S1. Details of electroadhesion (EA) experiments done with some of the animals shown in Figure 2. In each case, the photo shows the results of an EA experiment with a cationic (G^+) gel and the relevant animal. Adhesion is observed in all cases. In (A) and (B), adhesion is to the outer part of the animal. In (C) and (D), adhesion is to the inner soft tissue.

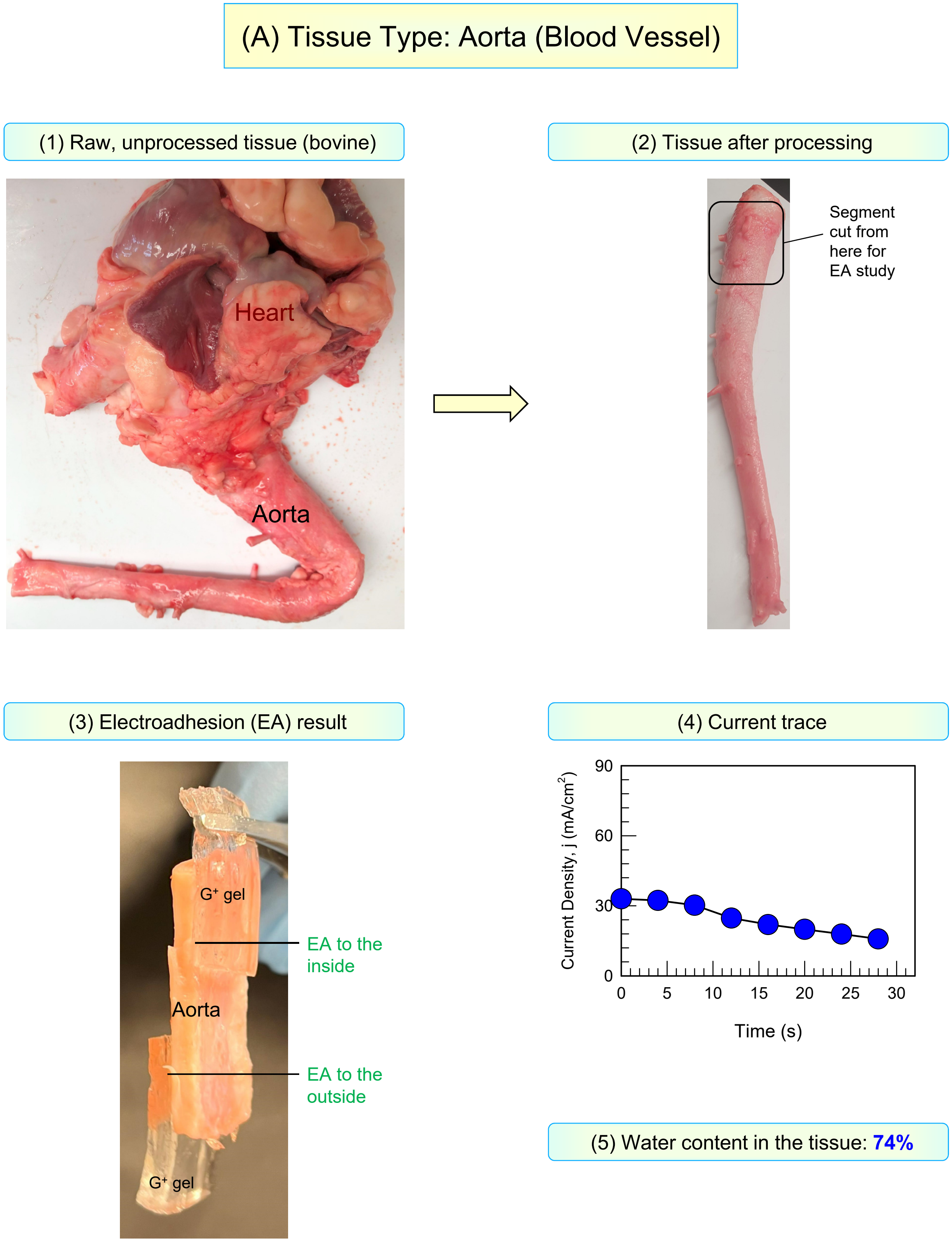

Figure S2A. Details of experiments with various mammalian tissues that exhibited electroadhesion (EA): Aorta. (1) Photo of the raw, unprocessed tissue. (2) Photo of the tissue after processing. (3) Photo of an EA experiment with a cationic (G^+) gel and a segment of the tissue. Strong adhesion is observed. (4) Trace of the current density (i.e., current/surface area) over the course of the EA experiment. (5) Water content in the tissue, measured by drying the tissue to constant weight at 50°C.

(B) Tissue Type: Trachea (Wind pipe)

(1) Raw, unprocessed tissue (bovine)

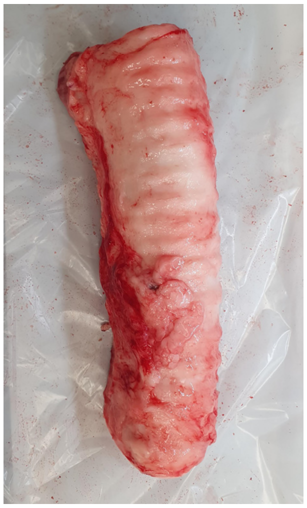

(2) Tissue after processing

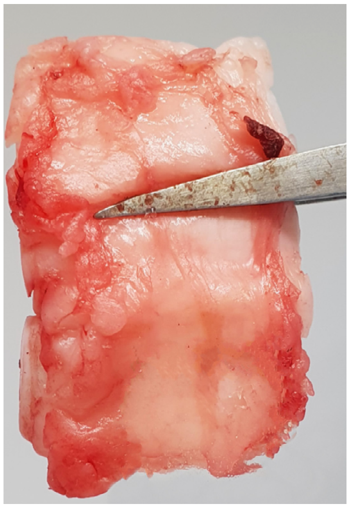

(3) Electroadhesion (EA) result

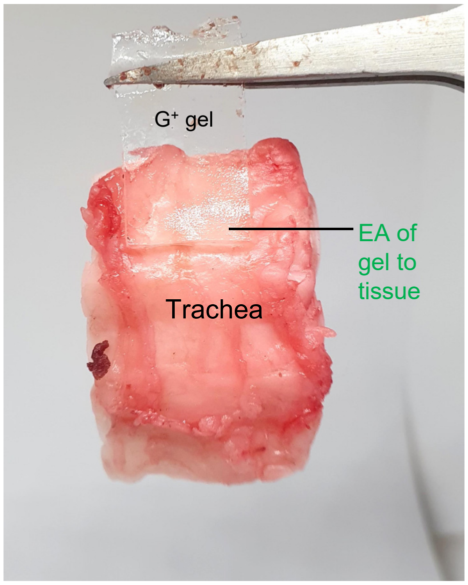

(4) Current trace

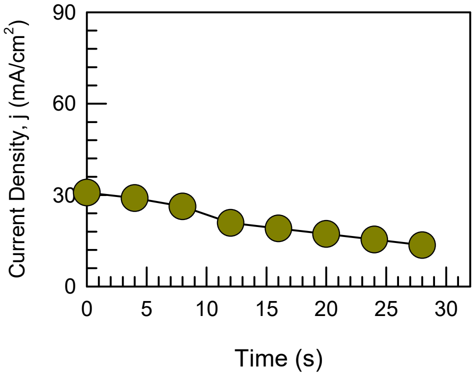

(5) Water content in the tissue: **58%**

Figure S2B. Details of experiments with various mammalian tissues that exhibited electroadhesion (EA): Trachea. (1) Photo of the raw, unprocessed tissue. (2) Photo of the tissue after processing. (3) Photo of an EA experiment with a cationic (G^+) gel and a segment of the tissue. Strong adhesion is observed. (4) Trace of the current density (current/surface area) over the course of the EA experiment. (5) Water content in the tissue, measured by drying the tissue to constant weight at 50°C.

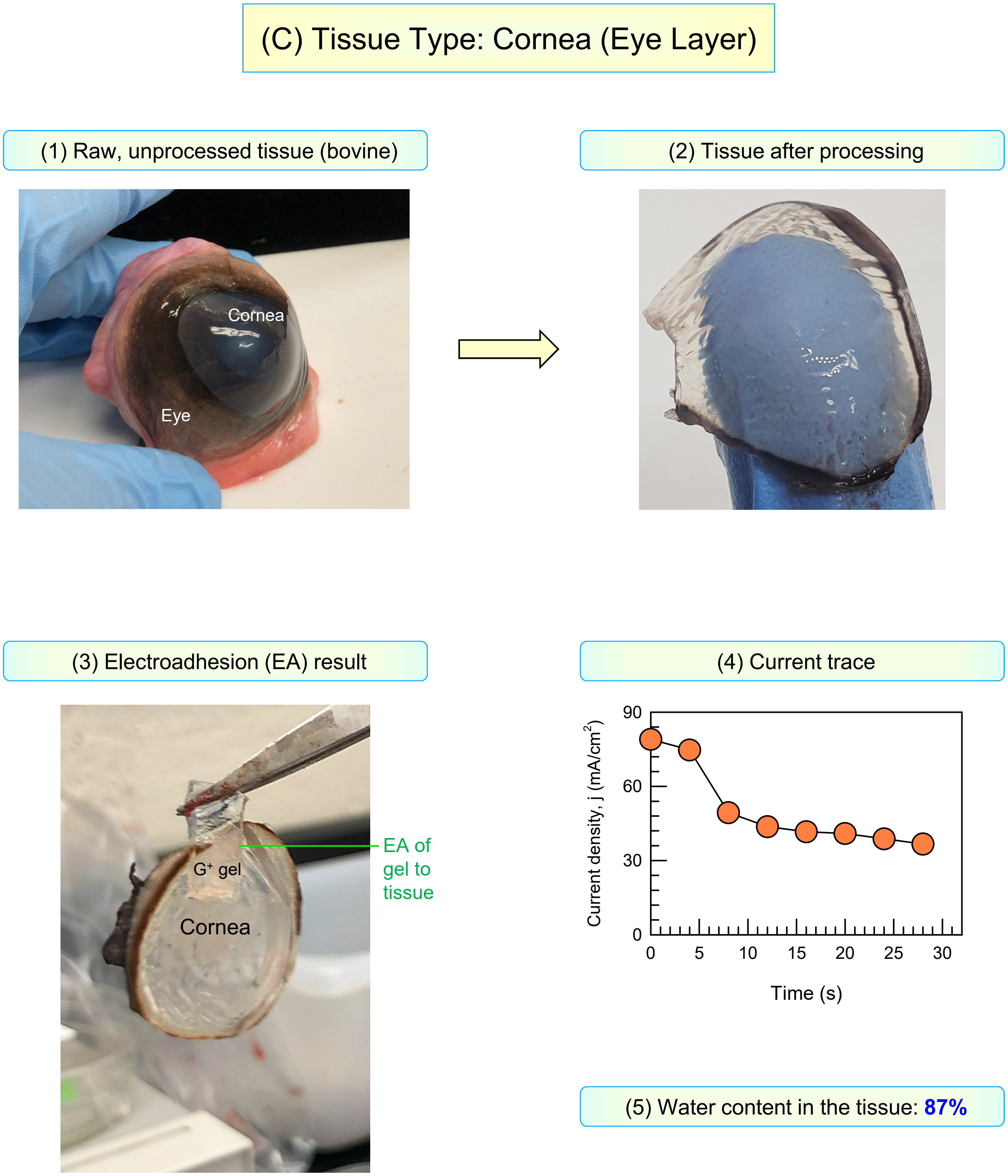

Figure S2C. Details of experiments with various mammalian tissues that exhibited electroadhesion (EA): Cornea. (1) Photo of the raw, unprocessed tissue. (2) Photo of the tissue after processing. (3) Photo of an EA experiment with a cationic (G^+) gel and the tissue. Strong adhesion is observed. (4) Trace of the current density (current/surface area) over the course of the EA experiment. (5) Water content in the tissue, measured by drying the tissue to constant weight at 50°C.

(D) Tissue Type: Heart

(1) Raw, unprocessed tissue (bovine)

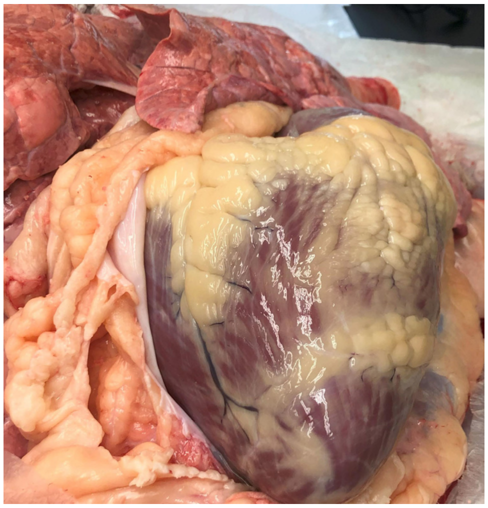

(2) Tissue after processing

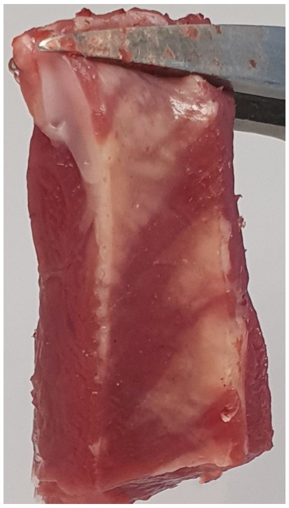

(3) Electroadhesion (EA) result

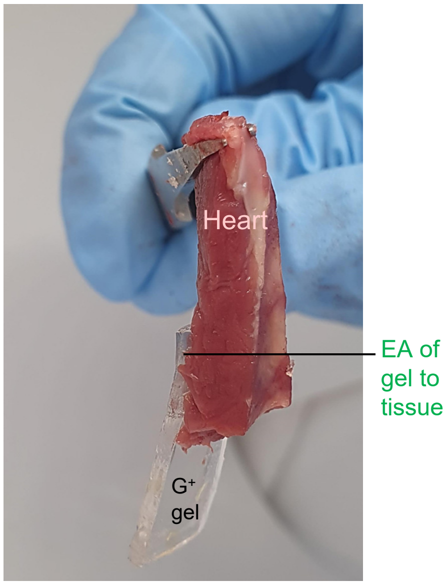

(4) Current trace

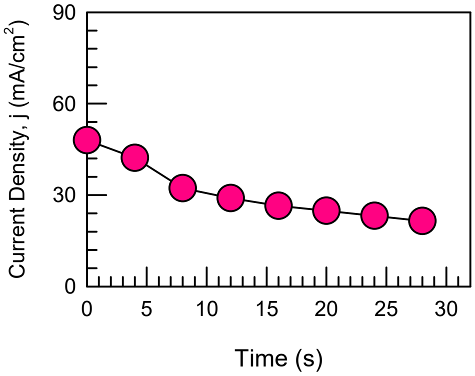

(5) Water content in the tissue: **76%**

Figure S2D. Details of experiments with various mammalian tissues that exhibited electroadhesion (EA): Heart. (1) Photo of the raw, unprocessed tissue. (2) Photo of the tissue after processing. (3) Photo of an EA experiment with a cationic (G^+) gel and a segment of the tissue. Moderately strong adhesion is observed. (4) Trace of the current density (current/surface area) over the course of the EA experiment. (5) Water content in the tissue, measured by drying the tissue to constant weight at 50°C.

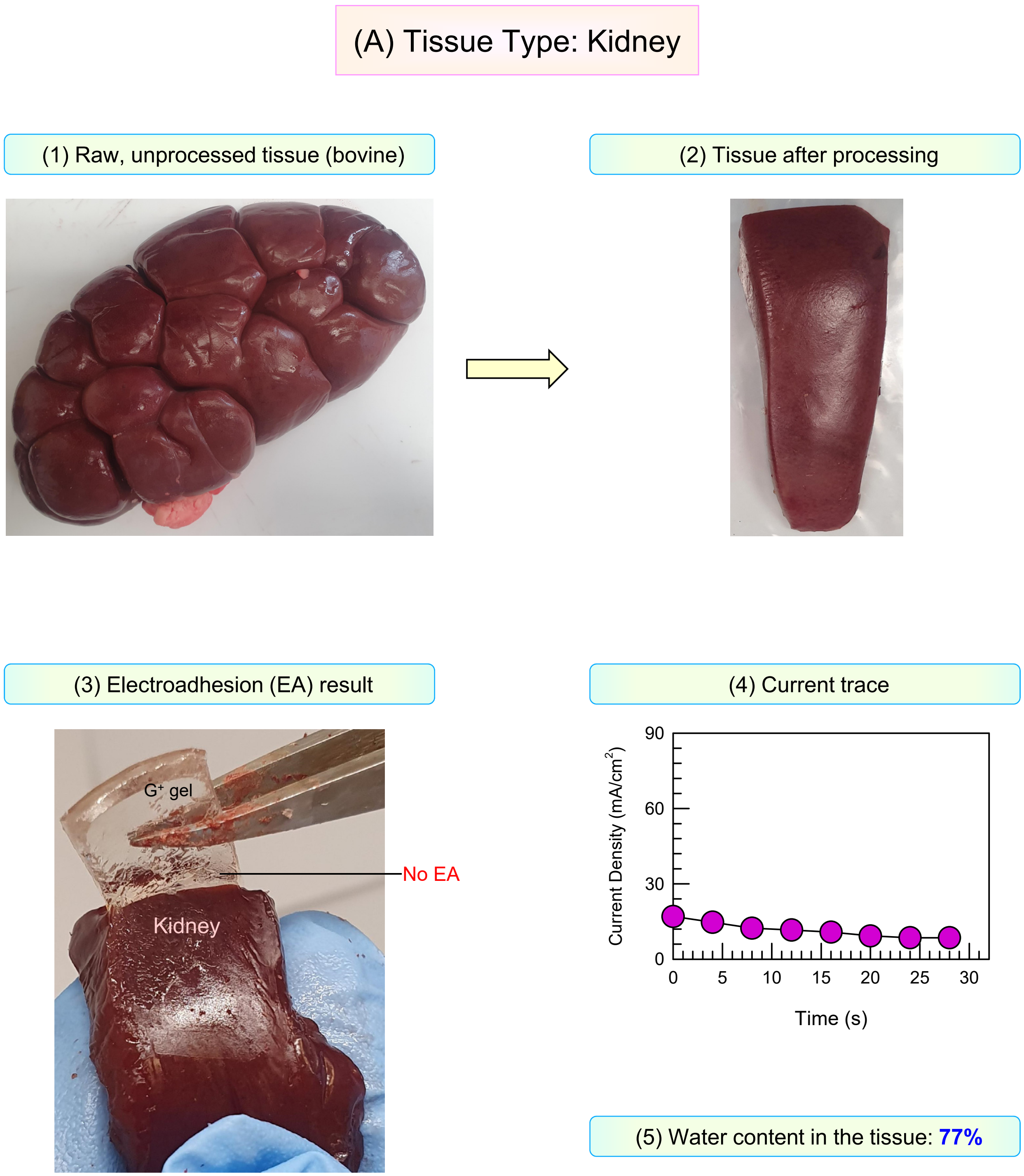

Figure S3A. Details of experiments with various mammalian tissues that did not exhibit electroadhesion (EA): Kidney. (1) Photo of the raw, unprocessed tissue. (2) Photo of the tissue after processing. (3) Photo of an EA experiment with a cationic (G^+) gel and a segment of the tissue. Adhesion is not observed. (4) Trace of the current density (current/surface area) over the course of the EA experiment. (5) Water content in the tissue, measured by drying the tissue to constant weight at 50°C.

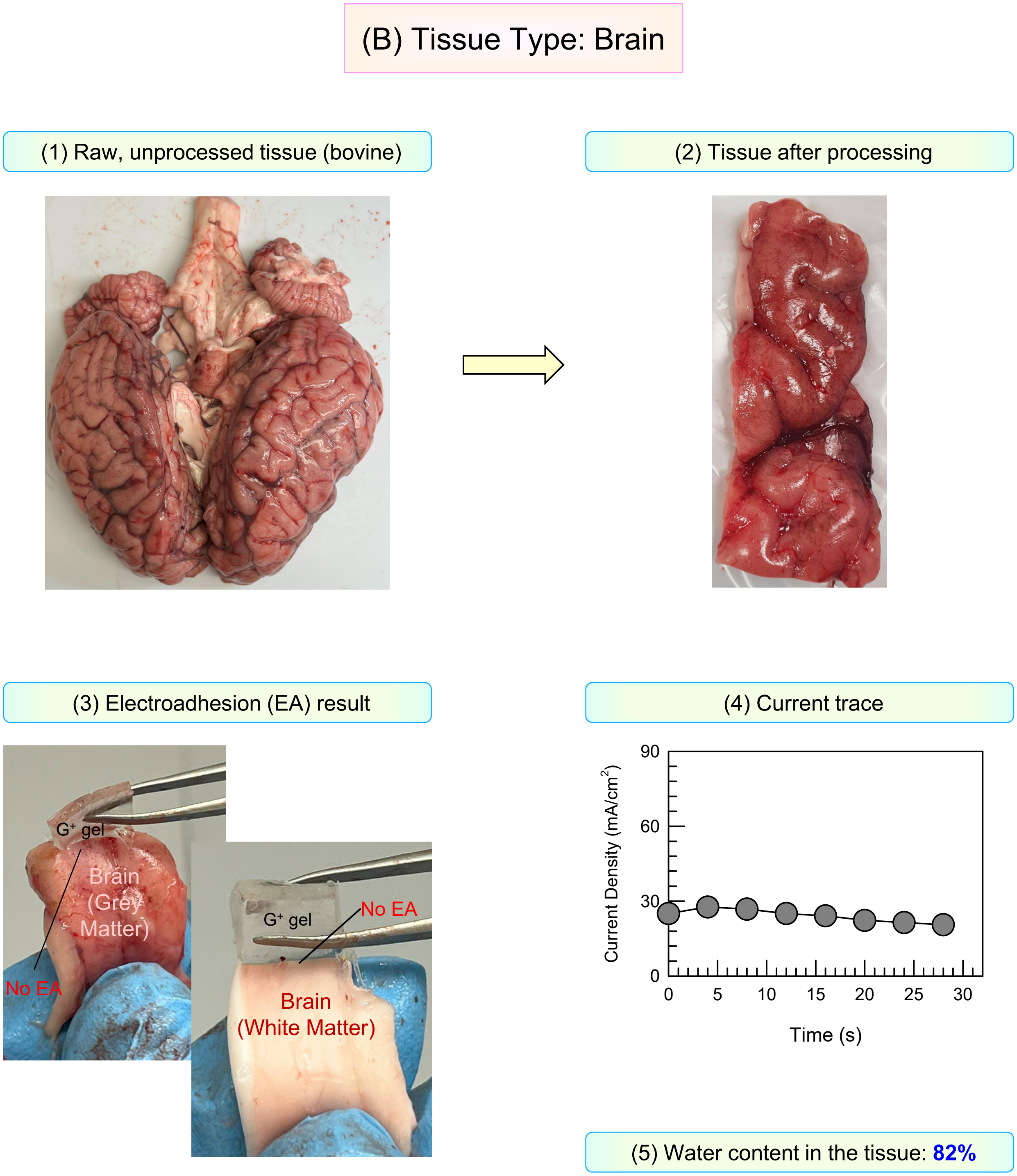

Figure S3B. Details of experiments with various mammalian tissues that did not exhibit electroadhesion (EA): Brain. (1) Photo of the raw, unprocessed tissue. (2) Photo of the tissue after processing. (3) Photos of EA experiments with a cationic (G^+) gel and segments of the tissue. Adhesion is not observed. (4) Trace of the current density (current/surface area) over the course of the EA experiment. (5) Water content in the tissue, measured by drying the tissue to constant weight at 50°C.

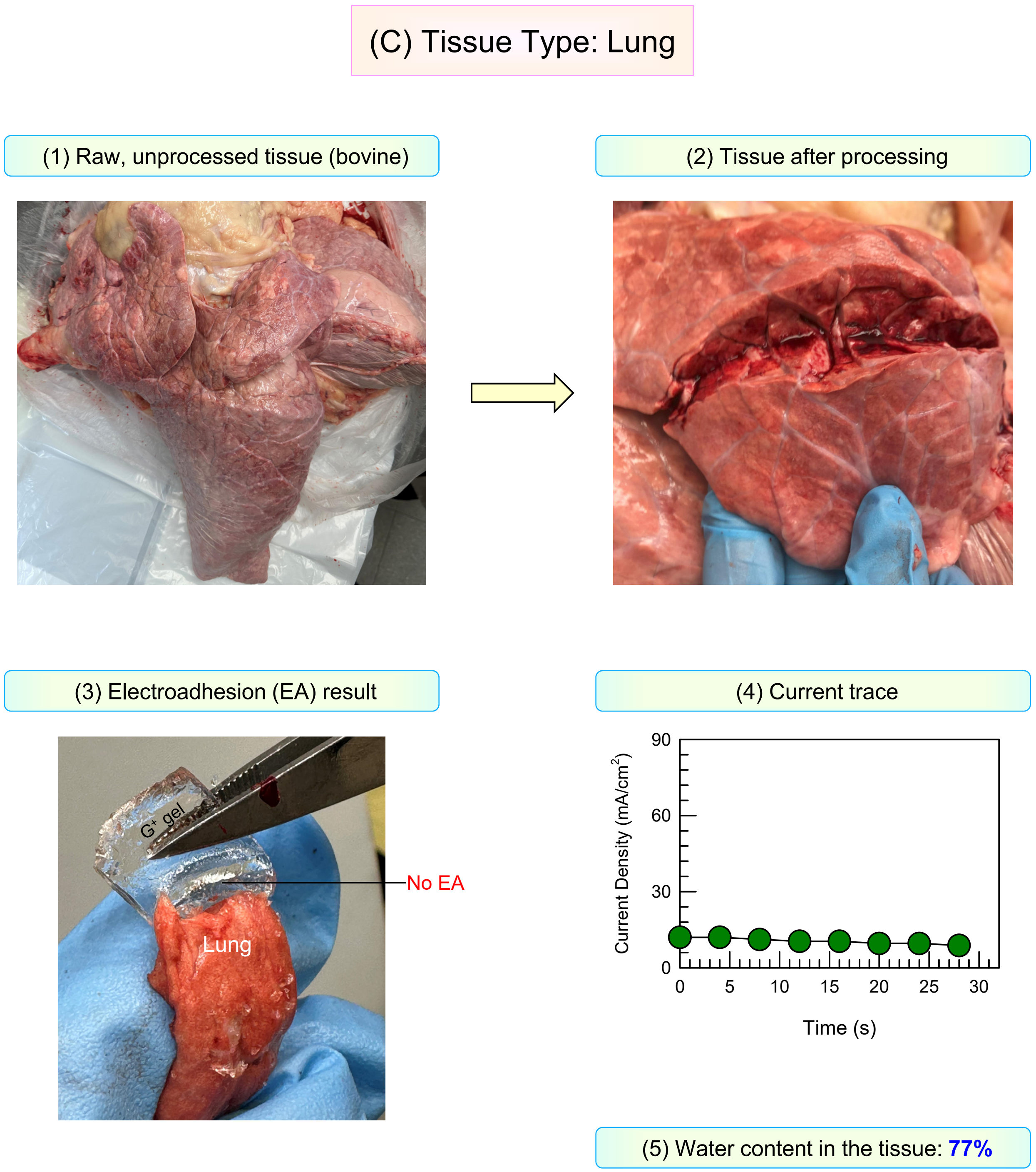

Figure S3C. Details of experiments with various mammalian tissues that did not exhibit electroadhesion (EA): Lung. (1) Photo of the raw, unprocessed tissue. (2) Photo of the tissue after processing. (3) Photo of an EA experiment with a cationic (G^+) gel and a segment of the tissue. Adhesion is not observed. (4) Trace of the current density (current/surface area) over the course of the EA experiment. (5) Water content in the tissue, measured by drying the tissue to constant weight at 50°C.

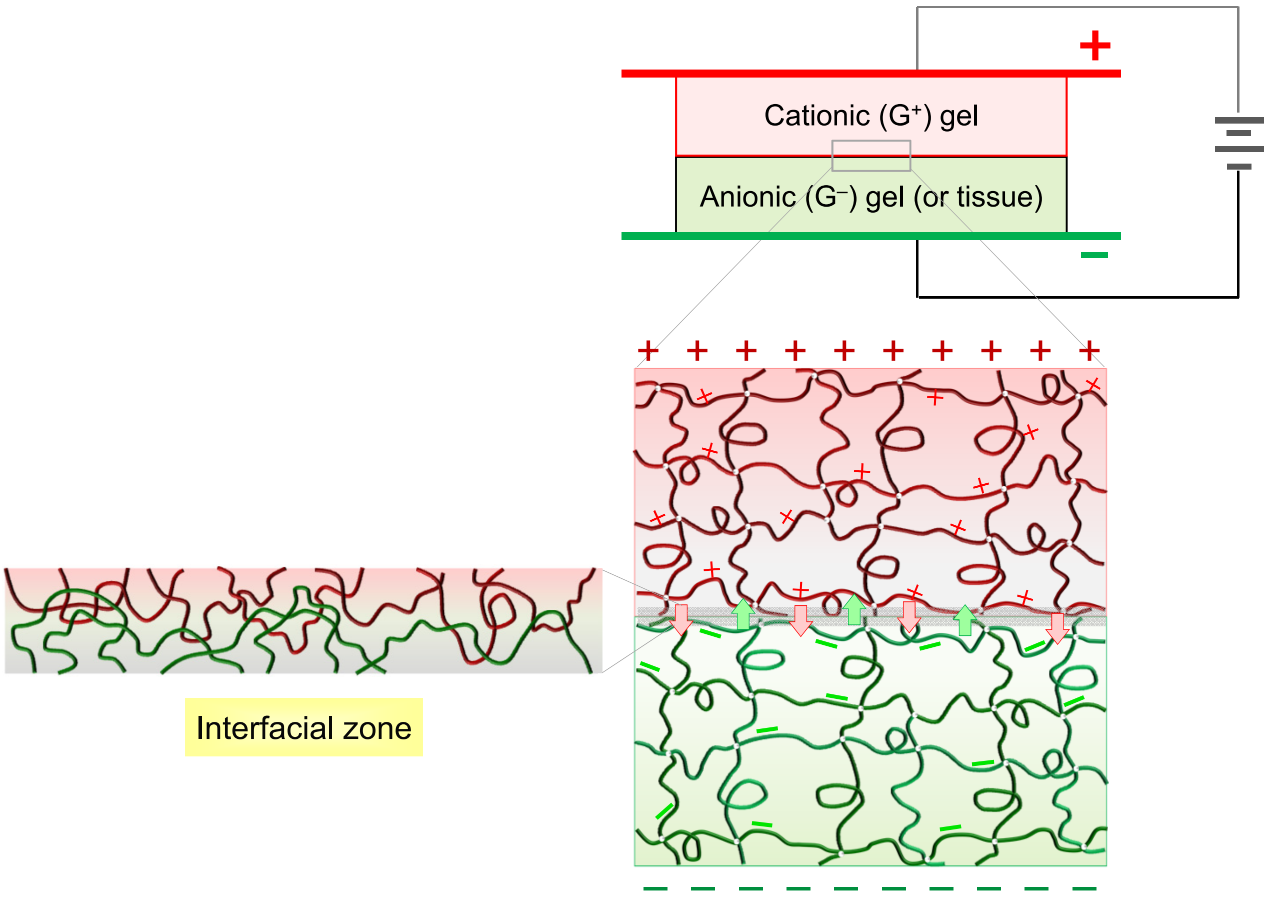

Figure S4. Mechanism hypothesized for electroadhesion (EA) between cationic and anionic materials. The mechanism is illustrated for the case of a cationic gel (G^+) and an anionic gel (G^-). The gels are connected to the power supply in the adhesion orientation (i.e., $E^+G^+T^-E^-$). When the field is switched on, cationic chains between crosslinks in the G^+ network undergo electrophoresis towards the anionic side. Likewise, anionic chains between crosslinks in the G^- network undergo electrophoresis towards the cationic side. This leads to the entanglement and binding of cationic and anionic chains in the interfacial zone between the gels, thereby inducing EA. When the field is switched off, the adhesion persists because of the strong binding of chains at the interface.

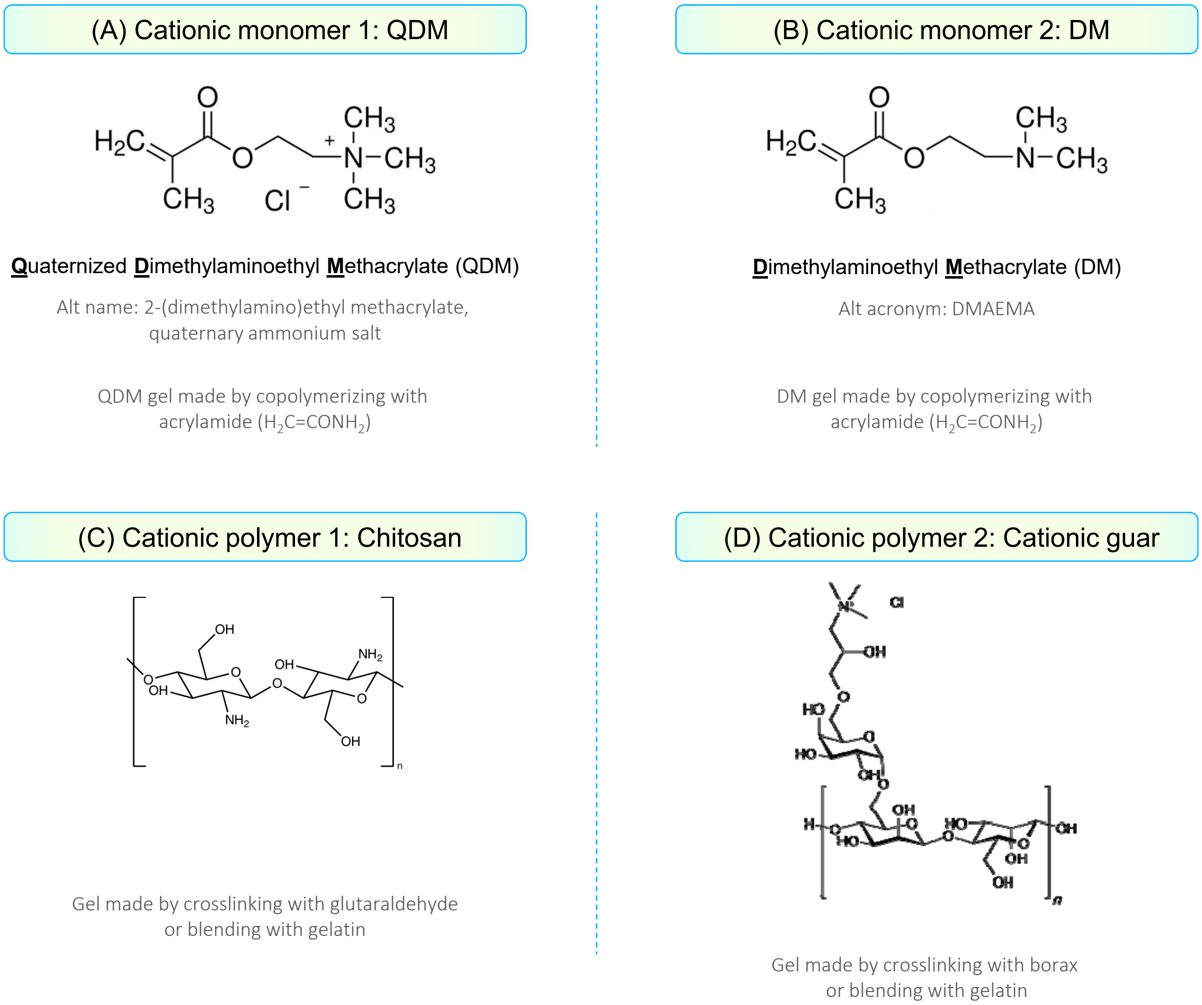

Figure S5. Chemistries of various cationic gels that can be adhered to tissues by electroadhesion. (A) and (B) are made by covalent crosslinking (polymerization) of cationic monomers. (C) and (D) are made by crosslinking linear polymers. (A) QDM gels are prepared by combining QDM (structure shown; note the cationic charge on the quaternary ammonium) and acrylamide (AAm) in water and polymerizing in the presence of a crosslinker. (B) Gels of the cationic monomer DM (structure shown) are made in a similar manner. (C) Gels of the cationic polymer chitosan (structure shown) are made by covalent crosslinking with glutaraldehyde. (D) Gels of cationic guar (structure shown) are made by physical crosslinking with borax. Gels of chitosan and cationic guar can also be made by simply blending the polymers into gels of a neutral protein like gelatin.
